# The SARS coronavirus papain like protease can inhibit IRF3 at a post activation step that requires deubiquitination activity

**DOI:** 10.1186/s12985-014-0209-9

**Published:** 2014-12-07

**Authors:** Krystal Matthews, Alexandra Schäfer, Alissa Pham, Matthew Frieman

**Affiliations:** Department of Microbiology and Immunology, University of Maryland at Baltimore, 685 West Baltimore St., Room 380, Baltimore, MD 21201 USA; Department of Epidemiology, University of North Carolina, 3304 Michael Hooker Research Building, Chapel Hill, NC 27599 USA; Department of Microbiology, Icahn School of Medicine at Mt. Sinai, New York, NY 10029 USA; Current Address: NYU Langone Medical Center, Department of Pathology, 538 Medical Science Building, New York, NY 10016 USA

**Keywords:** Coronavirus, Severe acute respiratory syndrome, IRF3, Interferon, Innate immunity

## Abstract

**Background:**

The outcome of a viral infection is regulated by complex interactions of viral and host factors. SARS coronavirus (SARS-CoV) engages and regulates several innate immune response pathways during infection. We have previously shown that the SARS-CoV Papain-like Protease (PLpro) inhibits type I interferon (IFN) by inhibiting IRF3 phosphorylation thereby blocking downstream Interferon induction. This finding prompted us to identify other potential mechanisms of inhibition of PLpro on IFN induction.

**Methods:**

We have used plasmids expressing PLpro and IRF3 including an IRF3 mutant that is constitutively active, called IRF3(5D). In these experiments we utilize transfections, chromatin immunoprecipitation, Electro-mobility Shift Assays (EMSA) and protein localization to identify where IRF3 and IRF3(5D) are inhibited by PLpro.

**Results:**

Here we show that PLpro also inhibits IRF3 activation at a step after phosphorylation and that this inhibition is dependent on the de-ubiquitination (DUB) activity of PLpro. We found that PLpro is able to block the type I IFN induction of a constitutively active IRF3, but does not inhibit IRF3 dimerization, nuclear localization or DNA binding. However, inhibition of PLpro’s DUB activity by mutagenesis blocked the IRF3 inhibition activity of PLpro, suggesting a role for IRF3 ubiquitination in induction of a type I IFN innate immune response.

**Conclusion:**

These results demonstrate an additional mechanism that PLpro is able to inhibit IRF3 signaling. These data suggest novel innate immune antagonism activities of PLpro that may contribute to SARS-CoV pathogenesis.

## Introduction

The severe acute respiratory syndrome coronavirus (SARS-CoV) emerged in 2002 from the Guangdong Province in China [1,2]. The disease quickly spread throughout the world, infecting more than 8,000 individuals and causing ~800 deaths. SARS-CoV caused significant economic losses around the world, as air travel was banned and/or limited in many affected regions. The epidemic strain of SARS-CoV is no longer circulating in the human population, however a strain of SARS-CoV potentially able to infect humans is still found in bats in China [3,4]. Additionally, many bat coronaviruses have been found around the world, including North America (Colorado [5], Maryland [6] and Canada [7]), Europe (Germany [8]) and Africa (South Africa [9]) that have the potential to become human pathogens. Importantly, a novel Coronavirus has emerged in the Middle East, called the Middle East respiratory syndrome coronavirus [10] (MERS-CoV) that has currently infected 853 individuals in 22 countries, including Saudi Arabia, Qatar and Jordan, resulting in 301 deaths as of October 2, 2014 (www.who.org). MERS-CoV is phylogenetically grouped into the beta-coronavirus lineage with SARS-CoV however it has been defined as a sub-group displaying unique genomic characteristics including unique accessory proteins [11]. The emergence of MERS-CoV has shown that, although SARS-CoV may not re-emerge directly, other coronaviruses are capable of emerging and causing significant respiratory illness in humans.

Relative to other respiratory viruses, SARS-CoV does not stimulate a robust innate immune response *in vitro* nor *in vivo* [12,13], perhaps explaining the significant lung disease caused by SARS-CoV in humans and mice in comparison to other human coronaviruses, which usually only cause minor respiratory symptoms. We, and others, have shown that SARS-CoV encodes several proteins that block virus sensing and type I IFN signaling pathways, resulting in a reduced innate immune response [14-24]. The inhibition of the host response to SARS-CoV leads to dampened production of host anti-viral proteins, and thus resulting in higher viral loads, more severe tissue damage, and enhanced lung pathology in mouse models of SARS-CoV [25].

PLpro is a domain of the larger, virally encoded replicase protein, called non-structural protein 3 or NSP3 [26]. PLpro cleaves specific sites in the ORF1ab polyprotein to release the replicase proteins from the longer polypeptide to facilitate SARS-CoV replication. The Papain-like Protease (PLpro) of SARS-CoV has been previously described to inhibit the type I IFN signaling pathway [16,18,19,23,27-30].

The induction of the innate immune response is key to protecting a host from viral infection [31]. In the IFN pathway, non-host RNA is sensed by several proteins including retinoic acid-inducible gene 1 (RIG-I) and melanoma differentiation-associated protein 5 (MDA5), which then signal through mitochondrial antiviral-signaling protein (MAVS) to activate IKK kinase epsilon (IKKi) and Tank binding kinase 1 (TBK1) [32]. IKKi and TBK1 phosphorylate IRF3, leading to its dimerization, import into the nucleus, and cooperation with other factors, to induce expression of IFNβ. IFNβ is secreted, binds to neighboring cells via the IFN alpha receptor I (IFNAR1), where it signals through the ISGF3 complex to induce several hundred anti-viral proteins that can fortify the cell’s response to infection.

In addition to PLpro’s protease activity, it has been shown to have deubiquitination and de-ISGylation activities [16,18,28,29,33]. Studies on PLpro have shown that it also inhibits host innate immune signaling by inhibiting phosphorylation, dimerization and nuclear import of IRF3 [16,18,28,29,33]. A recent report demonstrated that PLpro interacts with stimulator of IFN genes (STING), a scaffolding protein associated with the mitochondrial membrane that interacts with IRF3, RIG-I, IKKi and TBK1 [29]. By blocking phosphorylation of IKKi and TBK1, PLpro interaction with STING prevents the sensing of SARS-CoV RNA in the cell, and subsequent induction of IFNβ.

It has been shown previously that PLpro can block IRF3 phosphorylation [23]. We examined the inhibition of IRF3 after phosphorylation using a constitutively active phosphor-mimetic of IRF3, called IRF3(5D). We find that PLpro is able to inhibit IRF3(5D) even though IRF3(5D) can dimerize, be imported to the nucleus and bind several type I IFN inducible promoters. By mutating the active site of PLpro, we show that IRF3(5D) is no longer deubiquitinated and can now induce IFNβ gene production. These data demonstrate the multifunctional role of PLpro in inhibiting the innate immune response and suggests an additional role of PLpro during SARS-CoV infection.

## Materials and methods

### Plasmids and cells culture

Firefly luciferase plasmids containing the IFN-β or NF-κB promoter and the GFP- and HA-tagged SARS-CoV PLpro expression plasmids were described previously [16]. The SARS-CoV PLpro mutant used contains a double mutation in the active site (C1651A and D1826A) as described previously [17]. Flag-tagged IRF3(5D) was a gift from John Hiscott (described in[34]). Ha-tagged Ubiquitin was previously described [16]. HEK293T cells were purchased from ATCC (Catalog #CRL-3216) (Manassas, VA), grown in DMEM (Invitrogen, Carlsbad, CA) with 10% FBS and 1% penicillin/streptomycin.

### Luciferase assays

To analyze the induction of IFNβ induced genes, a luciferase reporter assay was used in HEK293T cells. Briefly, an expression construct containing the luciferase ORF and the IFNβ promoter (IFNβ/luciferase) was co-transfected with either a GFP control plasmid or the designated PLpro plasmid. Transfections of reporter plasmids into HEK293T cells were performed with the Lipofectamine LTX (Invitrogen) transfection reagent as directed by the manufacturer. For all transfections, 10 ng of Renilla luciferase, 200 ng of luciferase plasmid, 200 ng of viral expression plasmid, 200 ng of inducer plasmid (total 600 ng/well) was used in each well of a 48 well plate with 1ul of Lipofectamine LTX. For control wells that contained only the inducer and luciferase plasmid and did not contain 600 ng plasmids in total, the remaining DNA was filled with an empty GFP plasmid so we could assess transfection efficiency and so that all transfections contained the same amount of plasmid DNA. All transfections were performed in triplicate and the average of 3 experiments is shown in figures. At 18 hours post transfection cells were lysed and assayed for luciferase expression using the Dual Luciferase Reporter assay (Promega Inc). The ratio of experimental treatment to control inducer after normalization to Renilla luciferase was graphed in each figure.

### Ubiquitination and immunoprecipitations

Plasmids vectors containing HA and FLAG tags ligated to the C or N termini of the indicated ORFs were transfected into HEK293T cells as described below. After 18 hours of expression, cells were treated with lysis buffer (20 mM Tris/HCl pH 7.5, 150 mM NaCl, 1% NP-40), the extract was centrifuged for 10 minutes at 4°C, and the supernatant was removed. 25ul of washed EZ View Red Anti-FLAG M2 Affinity Gel beads (Sigma, St Louis MO, #F2426) were added to each extract and rotated overnight at 4°C. Extract was then washed 3 times with lysis buffer and re-suspended in SDS PAGE loading buffer before boiling and electrophoresis.

For ubiquitination or deubiquitination experiments, the protocol above was followed with the addition of HA-tagged ubiquitin (HA-Ub) or mutant ubiquitin plasmids. Mutant ubiquitin plasmids that are only able to be ubiquitinated at either K48 or K63 were added to the transfection experiments as described above. The mutant and wildtype ubiquitin plasmids were previously described [35].

### Western blotting and antibodies

Expression plasmids were assayed for protein expression by Western blotting. Lysates were then run on SDS-PAGE gels (NuPage, Invitrogen) and blotted to PVDF membrane (Invitrogen). Proteins were visualized using anti-GFP antibody (G1544, Sigma Aldrich), anti-HA (Sigma H3663), anti-Flag (Sigma F7425), HRP-conjugated secondary anti-rabbit antibody (NA934, GE Life Sciences) and HRP-conjugated secondary anti-rabbit antibody (NA931, GE Life Sciences).

### IRF3 Dimer gels

IRF3 dimer gels were performed as described in Iwamura et al. [36] with the following alteractions. All buffers were made 24 hours in advance and chilled overnight at 4°C. Tris/Glycine running buffer was supplemented with 0.3 g Sodium Deoxycholate. Native gels (Biorad Ready gel) was pre-run for 30 minutes at 150 V before loading samples. Native PAGE gels were run at 10 V for 2.5 hours before soaking gel in running buffer for 30 minutes and transferring to PVDF for 90 minutes. An IRF3 and IRF3(5D) sampled was boiled in SDS/PAGE loading dye for 5 minutes before loading as a monomer control. Proteins were visualized with anti-Flag antibody (Sigma F7425) and anti-HA antibody (Sigma H3663).

### Electrophoretic mobility shift assay

Binding assays were performed with ten micrograms of whole cell extracts incubated in a total volume of 15 μL with buffer containing 50 mM HEPES (pH 7.9), 10% glycerol, 200 mM KCl, 5 mM EDTA (pH 8), 1 mM MgCl_2_, 5 mM DTT, and 1 μg of poly (deoxyinosine-deoxycytidylic) acid sodium salt (Sigma-Aldrich) to eliminate non-specific binding. Samples were incubated on ice for 10 min, followed by the addition of 150,000 CPU of ^32^P-labeled DNA probe and 20 min of incubation at room temperature. Oligonucleotide probes corresponding to the ISREs of ISG15 and OAS were annealed to their complementary oligonucleoides using annealing buffer containing 100 mM NaCl and 50 mM HEPES (pH 7.6). Forward sequences for probes used:Oas1b ISRE: TTCCCGGGAAATGGAAACTGAAAGTCCCAT,ISG15 ISRE: GATCGGAAAGGGAAACCGAAACTGAAGCC. T4 PNK (New England Biolabs) was used to end-label annealed probes with γ32ATP. Samples were electrophoresed at 180 Volts in 0.5% Trisborate-EDTA buffer on a 5% native polyacrylamide gel composed of 49:1 acrylamide to bis-acrylamide. Gels were dried on Whatman paper at 80°C for 1 h and exposed by autoradiogram.

### Chromatin immunoprecipitations

Chromatin immunoprecipitation (ChIP) analysis was performed by using the Chromatin Immunoprecipitation (ChIP) assay kit (Millipore, 17–295) and previously described [25]. Briefly, HEK293T cells were transfected with 200 ng of each plasmid expressing either HA tagged PLpro, Flag tagged IRF3 or Flag tagged IRF3(5D) singly or in combination. At 24 hours post transfection, cells were fixed, pelleted and immediately frozen at −80°C. For immunoprecipitation the pellets were lysed and sonicated, with sonication conditions chosen to produce the desired size distribution of chromatin, between 300 and 1,200 bp. Lysates were immunoprecipitated with anti-IRF3 (Active Motif, 39033), anti-Flag (Sigma, F7425), anti-acetyl-Histone H3 (Millipore, 06–599) as a positive control, and anti-IgG (Jackson Labs, 315-005-003) as a negative control. To affirm the presence or absence of specific IRF3-binding to the IFNβ promoter following ChIP, PCR was performed. Response-specific IFNβ promoter regions were amplified by using the following primers: IFN-f 5’- GAATCCACGGATACAGAACCT-3’, IFN-r 5’-TTGACAACACGAACAGTGTCG-3’. The amplification of GAPDH served as an input control (forward 5’-CATGGGGAAGTTGAAGGTCG-3’, reverse 5’-TTGATGGTACATGACAAGGTGC-3’). PCR products were run on a 1.5% agarose gel for visualization.

### Immunofluorescence

GFP tagged PLpro, Flag tagged IRF3 and IRF3(5D) and GFP plasmids were transfected into HeLa cells as described above and cells fixed with 4% PFA. Fixed cells were incubated with mouse anti-Flag M2 antibody (Sigma-Aldrich, St. Louis MO). Cover-slips were incubated with secondary antibodies; Alexa Fluor 546 conjugated goat anti-mouse (Invitrogen, Carlsbad, CA). Fluorescence imaging was performed using a Zeiss Axioskop Microscope. The IRF3 and IRF3(5D) localization assays were performed in triplicate. Several fields of view were imaged for each transfection experiment and representative images consistent across each experiment displaying the localization of IRF3 and IRF3(5D).

## Results

### SARS-CoV PLpro inhibits constitutively active IRF3(5D)

SARS-CoV PLpro has been shown to inhibit IRF3 phosphorylation, nuclear translocation and subsequent IFNβ gene induction [16,23]. We sought to identify whether PLpro acts at additional downstream targets to inhibit IFNβ induction. To bypass the IRF3 phosphorylation step in the signaling pathway, we used a constitutively active phosphomimetic mutant of IRF3, called IRF3(5D). IRF3(5D) has 5 of the C-terminal serine residues mutated to aspartic acid (D), mimicking the C-terminal phosphorylated serine of active IRF3, bypassing the need for its phosphorylation [34]. We hypothesized that, as PLpro inhibits IRF3 phosphorylation, IRF3(5D) would not have any effect on the downstream activation of STING/IKK/IRF3 and allow us to analyze the effects of PLpro on IRF3 signaling after its phosphorylation step.

**Figure 1:**
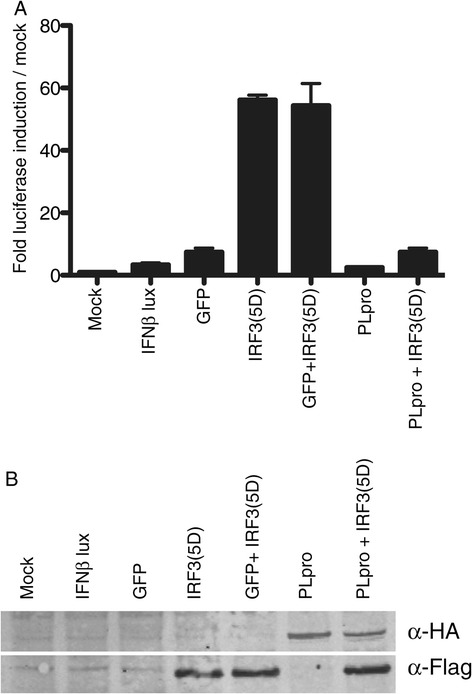
**SARS-CoV PLpro inhibits IRF3(5D) induction of IFNβ. A**. Plasmid expressing SARS-CoV PLpro was tested for the ability to inhibit the induction of an IFNβ promoter/luciferase reporter induced by IRF3(5D). The average induction with standard deviation across 3 transfections is graphed relative to mock transfection. HEK293T cells were transfected with plasmids encoding IFNβ promoter/luciferase, Flag-tagged IRF3(5D), empty GFP vector or HA-tagged SARS-CoV PLpro in the combinations noted. At 18 hours post-transfection cells were lysed and firefly luciferase expression quantified. **B**. Lysate from each transfection in A was analyzed by western blot to assay protein expression of HA-tagged PLpro and Flag-tagged IRF3(5D).

To investigate this hypothesis, HEK293T cells were transfected with the IFNβ/luciferase plasmid alone, or in combination with a plasmid encoding IRF3(5D) (a potent inducer of the IFNβ promoter) and either an empty expression plasmid or a plasmid expressing a HA-tagged PLpro of SARS-CoV (Figure 1). We find that IRF3(5D) induces ~60-fold higher levels of luciferase compared to mock transfected cells and an empty GFP plasmid has no effect on luciferase expression, as expected. However, expression of HA-tagged PLpro caused a strong inhibition of IRF3(5D) dependent luciferase production. These data demonstrates that PLpro is able to inhibit IRF3 induced IFNβ gene induction at a post-phosphorylation step.

### SARS-CoV PLpro does not inhibit IRF3(5D) dimerization

**Figure 2:**
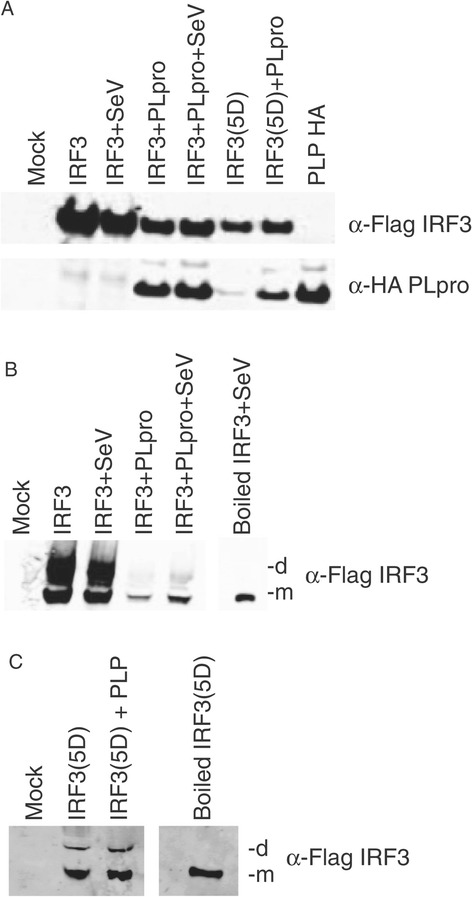
**SARS-CoV PLpro does not inhibit IRF3(5D) dimer formation.** The ability of SARS-CoV PLpro to inhibit IRF3(5D) dimer formation was assayed using native PAGE gels. **A**. HEK293T cells were transfected with either Flag-tagged IRF3, HA-tagged PLpro or Flag-tagged IRF3(5D) in combination or alone. For Flag-tagged IRF3 transfections, SeV was added at 18 hours post transfection for 6 hours before cells were lysed. 5ul of total lysate was run on a SDS/PAGE gel to assay input lysate levels. **B**. Lysate from transfections in A were ran on Native PAGE gels for IRF3 dimer visualization in the noted combinations of transfected plasmids, including a Flag-tagged IRF3 + SeV infected sample that was boiled before loading to show monomer size. **C**. Lysate from transfections in A were ran on Native PAGE gels for IRF3(5D) dimer visualization in the noted combinations of transfected plasmids, including a Flag-tagged IRF3(5D) that was boiled before loading to show monomer size. d = dimer, m = monomer.

Upon phosphorylation, IRF3 homodimerizes before entering the nucleus to induce IFN production. We hypothesized that SARS-CoV PLpro may be blocking IRF3(5D) dimerization to inhibit IFNβ induction. To this end, HEK293T cells were transfected with Flag-tagged IRF3 with and without SARS-CoV PLpro, and then infected with Sendai Virus (SeV), a potent inducer of IRF3 activation and IFNβ induction, for six hours before all cells were lysed and dimer formation assessed by SDS-PAGE (Figure 2A) and native PAGE gel electrophoresis (Figure 2B). As expected, in mock-infected cells, IRF3 is found as a dimer, which is not changed during SeV infection (Figure 2B) [37]. This is expected due to the previous finding that overexpression of IRF3 alone can induce IFNβ gene induction. However, when HA-tagged PLpro is co-transfected with IRF3, dimer formation is inhibited (Figure 2B). This confirms the previous finding that PLpro inhibits IRF3 phosphorylation, and thus its dimerization [23].

We performed the same assay on IRF3(5D)-transfected cells, with and without HA-tagged PLpro. We performed native PAGE on transfected HEK293T cells with a plasmid expressing Flag-tagged IRF3(5D) alone or in combination with HA-tagged PLpro (Figure 2C). We found that IRF3(5D) readily dimerizes when over-expressed alone, and PLpro has no effect on IRF3(5D) dimerization. These data demonstrate that the inhibition of IRF3(5D)-dependent IFNβ gene induction by PLpro is not due to PLpro blocking IRF3(5D) dimerization.

### PLpro does not block IRF3(5D) nuclear import

**Figure 3:**
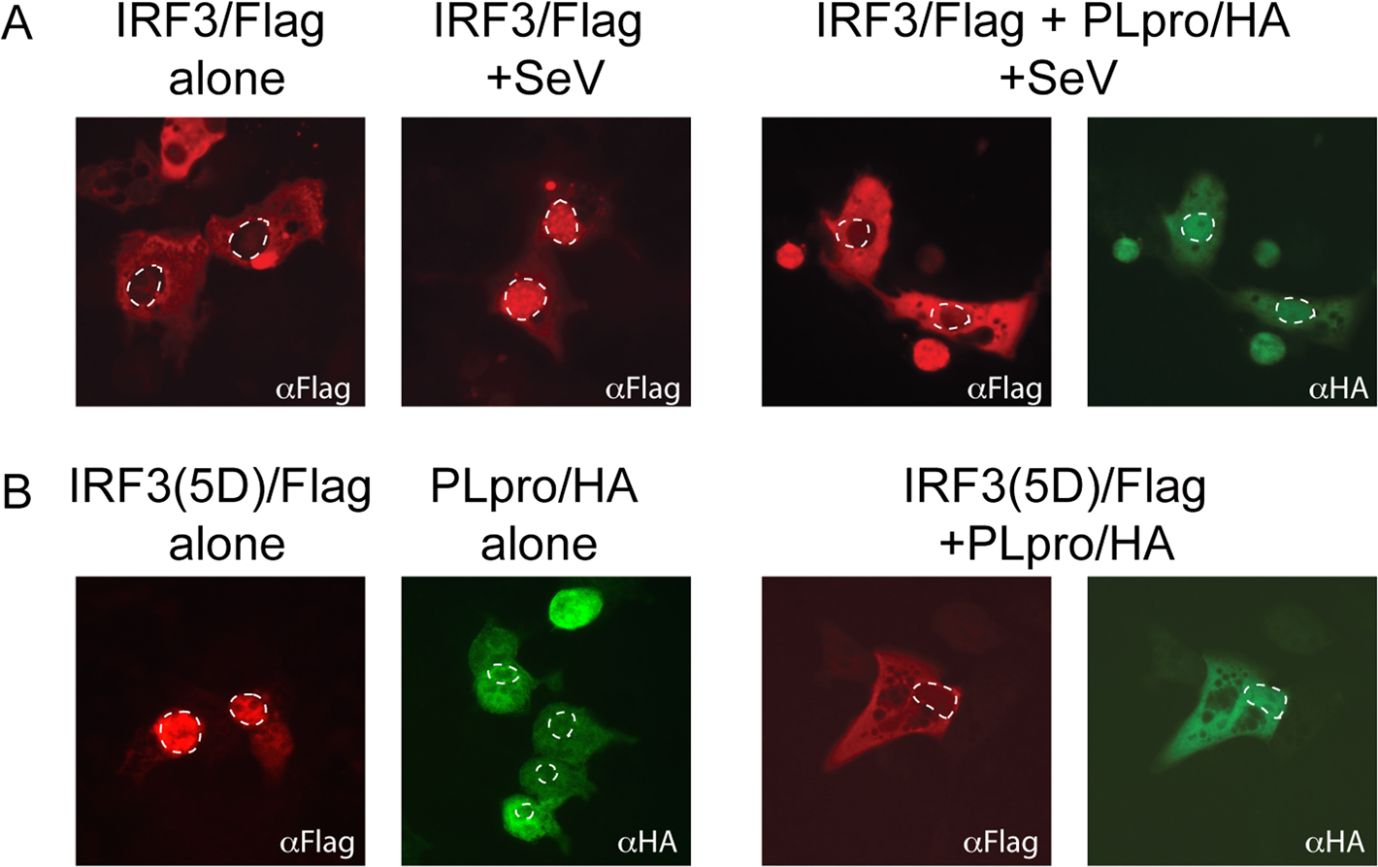
**Effect of SARS-CoV PLpro on localization of IRF3 and IRF3(5D).** Plasmids expressing either Flag-tagged IRF3, Flag-tagged IRF3(5D) or HA-tagged PLpro were transfected alone or in combination into HeLa cells and visualized with anti-Flag or anti-HA antibody. **A**. Flag-tagged IRF3 alone (left) or after SeV infection (middle), Flag-tagged IRF3 co-transfected with HA-tagged PLpro after SeV infection (right) **B**. Flag-tagged IRF3(5D) alone (left), HA-tagged PLpro alone (middle) and Flag-tagged IRF3(5D) co-transfected with HA-tagged PLpro. All cells fixed in 4% PFA and visualized with Alexa 488 conjugated anti-rabbit or Alexa 594 conjugated anti-mouse antibodies. Nuclei are delineated by white dashed lines.

The next step after dimerization in IRF3 signaling is nuclear import. We, therefore, hypothesized that PLpro is blocking nuclear import of IRF3(5D). To test this, we transfected Vero E6 cells with either Flag-tagged IRF3 and IRF3(5D) with or without HA-tagged PLpro. As a positive control, Flag-tagged IRF3 was transfected alone and at 24 hours post transfection cells, were infected with SeV for six hours. As expected, we found that prior to SeV infection, IRF3 is localized primarily in the cytoplasm, and migrates to the nucleus upon SeV infection (Figure 3A). When IRF3 and PLpro were co-transfected in the presence of SeV, we found that IRF3 is only in the cytoplasm. This demonstrates that PLpro most likely blocks nuclear import of activated IRF3 (Figure 3A).

IRF3(5D) localized to both the nucleus and the cytoplasm in transfected cells (Figure 3B), as previously reported [34], reflecting the continuous cytoplasmic translation and nuclear import IRF3(5D). When PLpro is over-expressed, IRF3(5D) is found in both the nucleus and the cytoplasm, identical to what is found when IRF3(5D) is expressed alone (Figure 3B). Therefore, we do not see a block of nuclear import of IRF3(5D) when PLpro is expressed. These data demonstrate that PLpro is not blocking IRF3(5D) nuclear import to inhibit IFNβ gene induction.

### PLpro does not block IRF3(5D) DNA binding activity

After activated IRF3 dimers are imported into the nucleus, they bind to specific response elements in the promoter region of IRF3-induced genes. Since PLpro does not block IRF3(5D) nuclear import, we hypothesized that it may block the ability of IRF3(5D) to bind to DNA promoters. We hypothesized that PLpro may be inhibiting the ability of IRF3 to bind to the DNA directly. Because PLpro is not found as a soluble protein in infected cells, rather it is a domain of the larger NSP3 protein, we would not hypothesize that PLpro would be binding DNA natively. However, it is used as a comparator for IRF3 binding to the promoter regions. In these experiments, we assayed the ability of PLpro to affect IRF3 binding to DNA using two assays. First, we tested for the ability of IRF3 to bind to the IFNβ promoter by performing chromatin immuno-precipitation (ChIP) and second, we assayed for the ability of co-transfected PLpro to inhibit IRF3 binding to other IFN-inducible promoters by gel-mobility shift assay.

**Figure 4:**
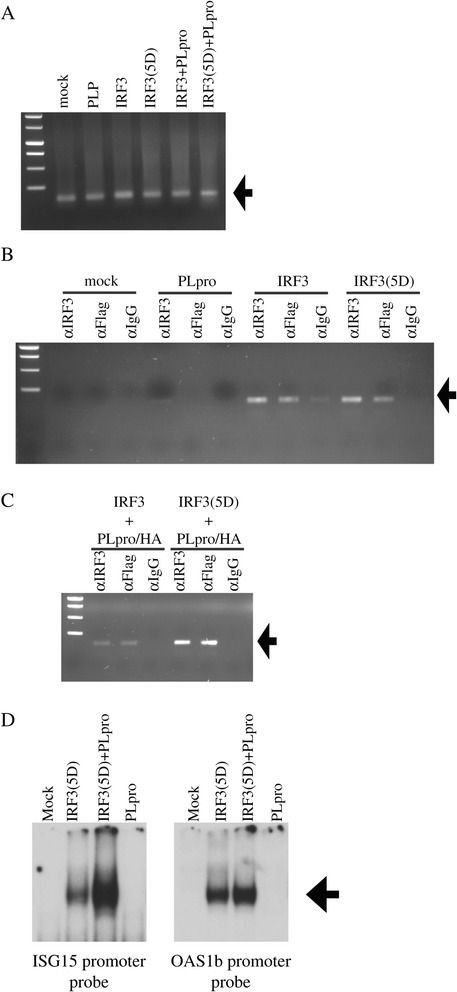
**SARS-CoV PLpro does not inhibit IRF3 or IRF3(5D) binding to the IFNβ promoter. A**-**C**. Chromatin immunoprecipitations (ChIP) were performed on HEK293T cells transfected with either HA tagged PLpro, Flag-tagged IRF3, Flag-tagged IRF3(5D) or the noted combinations. At 24 hours post transfection, cells were fixed with 4% Paraformaldehyde and DNA was extracted and sonicated to ~300-1.2Kb in length. Samples were then immunoprecipitated with the designated antibodies and used as template for PCR reactions for GAPDH to control for input template (**A**, arrow denotes correct PCR product) of the IFNβ promoter (**B** and **C**, arrows denote correct PCR products). PCR reactions were run on 2% agarose gels to visualize products. **D**. HEK293T cells were transfected with either Flag-tagged IRF3(5D) alone or in combination with HA-tagged PLpro. Cells were lysed and lysates incubated with radioactively labeled probe from either the ISG15 promoter or the OAS1b promoter. Samples were run on a polyacrylamide gel and protein complexes visualized on film. Arrow denotes supershifted complex.

First, we performed ChIP of IRF3(5D) to identify whether PLpro was altering the ability of IRF3(5D) to bind the IFNβ promoter. HEK293T cells were transfected with Flag-tagged IRF3 or Flag-tagged IRF3(5D) with or without HA-tagged PLpro. At 24 hours post transfection, cells were treated with paraformaldehyde (PFA) to cross-link protein bound to DNA, lysed and sonicated to shear protein bound chromatin for ChIP experiments. Immunoprecipitation was performed against either IRF3, Flag or IgG (negative control). The pre-ChIP input was scored for GAPDH to control for the amount of template in the reactions (Figure 4A). After immunoprecipitating the samples, the crosslink was reversed and used for PCR with IFNβ promoter primers spanning the IRF3 binding site (Figure 4B). Negative controls of either untransfected protein or HA-tagged PLpro transfected alone, showed no amplification of the IFNβ promoter in immunoprecipitated samples, demonstrating no background binding of PLpro to the IFNβ promoter (Figure 4B). As expected, IFNβ promoter binding is observed in cells transfected with either IRF3 or IRF3(5D) (Figure 4B, at arrow). To assess the effect of PLpro on IRF3 binding activity, HA-tagged PLpro was co-transfected with either Flag-tagged IRF3 or Flag-tagged IRF3(5D). We found strong amplification of the IFNβ promoter in each case (Figure 4C), suggesting that PLpro had no effect on IRF3 or IRF3(5D) binding to the IFNβ promoter. These data demonstrate that the inhibition of IRF3(5D) by PLpro is not due to inhibition of IRF3(5D) binding to the IFNβ promoter.

Electro-mobility Shift Assays (EMSA) were used to test whether IRF3(5D) from cells containing both IRF3(5D) and PLpro was able to bind to the ISG15 or OAS1b promoter, both of which are bound by IRF3 during infection. In this assay, HEK293T cells were transfected with either empty plasmid, Flag-tagged IRF3(5D), HA-tagged PLpro or Flag tagged IRF3(5D) and HA-tagged PLpro together. Protein lysates were incubated with radiolabeled oligonucleotide probes that correspond to the ISG15 and OAS1 promoters. The negative control lysate from HA tagged PLpro transfected cells did not bind to either probe. However, supershifts, designating a protein-DNA complex, were visible for both ISG15 and OAS1 promoter elements in lysates containing IRF3(5D) (Figure 4D). Interestingly, lysate from cells containing both HA-tagged PLpro and Flag-tagged IRF3(5D) still bound to the promoter elements of both genes (Figure 4D). These data suggest that PLpro does not affect the ability of activated IRF3 to bind its DNA targets.

### IRF3(5D) activity is dependent on the de-ubiquitination activity of PLpro

**Figure 5:**
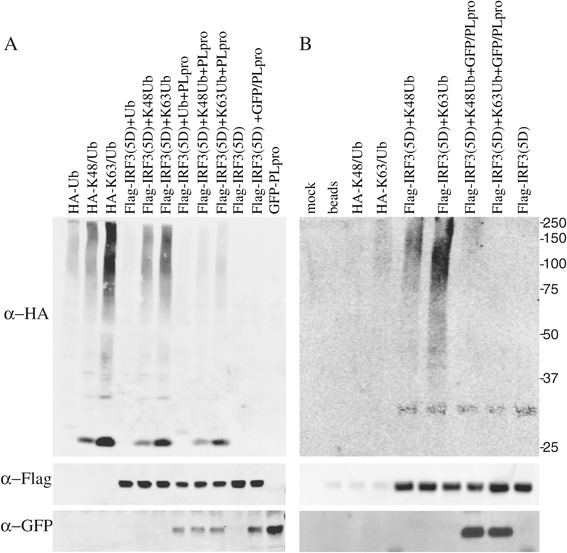
**IRF3(5D) is deubiquitinated by SARS-CoV PLpro.** Plasmids expressing either Flag-tagged IRF3(5D), GFP-tagged PLpro, HA-tagged Ubiquitin, HA-tagged K48 Ubiquitin, or HA-tagged K63 Ubiquitin were transfected into HEK293T cells alone or in combination to assay for ubiquitination statues of IRF3(5D). At 24 hours post transfection, cells were lysed and analyzed by western blot with anti-HA, anti-Flag and anti-GFP. **A**. A sample (5ul) of each indicated transfection was analyzed by western blot to detect input expression levels. **B**. Lysate was used for immunoprecipitation with antibody directed against the Flag tag to identify the ubiquitination state of IRF3(5D). Note that IRF3(5D) is ubiquitinated when transfected with HA-tagged K48 or K63 Ubiquitin, however when PLpro is cotransfected with as well, ubiquitination is reduced.

IRF3 has not been conclusively shown to be ubiquitinated. Other proteins in the IRF3 signaling pathway, such as RIGI-I [38], are ubiquitinated and can effect IRF3 activation. We examined whether ubiquitination of IRF3 is necessary for IRF3- dependent induction of IFNβ. We then sought to identify whether the ubiquitination of IRF3(5D) was affected by PLpro. PLpro contains de-ubiquitination (DUB) enzymatic activity and we tested whether IRF3(5D) was de-ubiquitinated by PLpro. We transfected HEK293T cells with either wildtype ubiquitin (Ub) tagged with HA (HA-Ub), K48-linked Ub (K48-Ub), or K63-linked Ub (K63-Ub) with and without Flag-tagged IRF3(5D) (Figure 5A). As expected, we observed a ladder of ubiquitinated proteins when probed with anti-HA in the lanes containing IRF3(5D) with either of the HA-tagged Ub variants or HA-tagged Ub variants alone. When Flag-tagged IRF3 and HA-tagged Ub were co-transfected with GFP-tagged PLpro we found that overall ubiquitinated protein levels were reduced (Figure 5A). Interestingly, K48-Ub and K63-Ub plasmid transfections displayed higher levels of ubiquitinated proteins compared to wildtype HA-Ub. For this reason, K48-Ub and K63-Ub were solely used in immunoprecipitation experiments in Figure 5B. However, wildtype HA-Ub does show reduced ubiquitinated protein levels when PLpro is co-expressed, identical to the results with K48-Ub and K63-Ub plasmid transfections. When anti-Flag coated beads were used to pull-down Flag-tagged IRF3(5D) from selected lysate shown in Figure 5A, we found that IRF3(5D) is ubiquitinated. Interestingly, when co-transfected with PLpro and K48 and K63 ubiquitin, there is only minor ubiquitination of IRF3(5D) (Figure 5B). These data demonstrate that IRF3(5D) is ubiquitinated and that PLpro is able to deubiquitinate IRF3(5D) *in vitro*.

**Figure 6:**
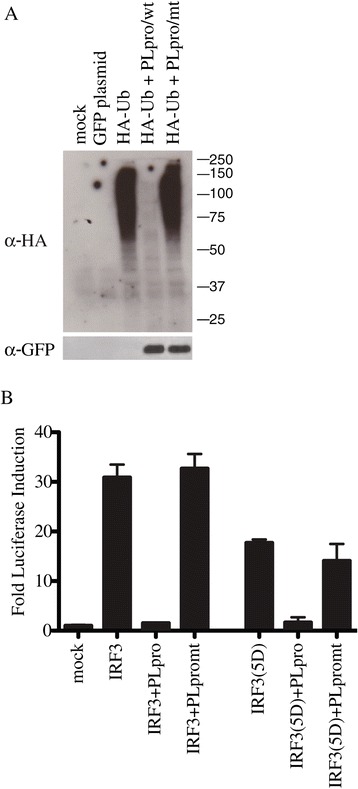
**IRF3(5D) activity is dependent on the de-ubiquitination activity of PLpro. A**. HEK293T cells were transfected with either empty plasmid, HA-tagged Ubiquitin alone or HA-tagged Ubiquitin in combination with either wildtype PLpro (PLpro/wt) or active site mutant PLpro (PLpro/mt). At 24 hours post-transfection, lysate was extracted and analyzed by western blot with anti-HA and anti-GFP antibodies to confirm that the PLpro/mt was unable to deubiquitinate cellular proteins. **B**. Plasmid expressing SARS-CoV PLpro/wt and PLpro/mt were tested for the ability to inhibit the induction of an IFNβ promoter/luciferase reporter induced by IRF3(5D). The average induction with standard deviation across 3 transfections is graphed relative to mock transfection. HEK293T cells were transfected with plasmids encoding IFNβ promoter/luciferase, Flag-tagged IRF3(5D), empty GFP vector, HA-tagged SARS-CoV PLpro/wt or PLpro/mt in the combinations noted. At 18 hours post-transfection cells were lysed and firefly luciferase expression quantified.

We hypothesized that the ubiquitination of IRF3(5D) was necessary for its activity, as has been shown for IRF3 [39], so we mutated the active site of PLpro to remove its deubiquitination activity [28]. To confirm the inhibition of the DUB activity, HEK293T cells were transfected with either a GFP-only plasmid, HA-tagged Ub alone or HA-tagged Ub in combination with either wild type PLpro (PLpro/wt) or active site mutant PLpro (PLpro/mt) (Figure 6A). As before, both PLpro plasmids are expressed as C-terminal GFP fusion proteins. We observe that when HA-tagged Ub is transfected alone, a large smear of ubiquitinated proteins are seen by western blot. When GFP-tagged WT PLpro was co-transfected with HA-tagged Ub, we observe a marked reduction in the amount of ubiquitinated proteins. When the mutant GFP-tagged PLpro is co-transfected with HA-tagged Ub the smear of ubiquitinated proteins reappeared (Figure 6A), demonstrating that the mutant PLpro no longer contains deubiquitinase activity.

We used this mutant PLpro to assay for its ability to inhibit IRF3(5D)-dependent induction of IFNβ/luciferase reporter plasmid. As seen previously, when either IRF3 or IRF3(5D) are transfected into HEK293T cells with the IFNβ/lux reporter we observe strong induction of luciferase (32-fold and 21-fold, respectively) (Figure 6B). Also, as observed previously, when wildtype PLpro is co-transfected with both we find that IFNβ/lux induction is significantly inhibited. However, when the deubiquitination- deficient mutant PLpro is transfected with either IRF3 or IRF3(5D) we found that there is no longer an inhibition of IFNβ/lux induction. These data demonstrate that the deubiquitination activity of PLpro is responsible for limiting gene induction of IRF3(5D) on the IFNβ promoter.

## Discussion

The innate immune response is the first pathway in the cell to detect a virus during infection and many viruses express proteins that actively inhibit this response [31,40]. SARS-CoV expresses several proteins that inhibit various innate immune sensing and response pathways [14-24]. We and others have shown that SARS-CoV PLpro blocks the innate sensing pathway by inhibiting IRF3 activation [16,23] through binding to STING [29]. In this work, we show that PLpro can act downstream of STING to block IRF3’s function as a transcription factor in the nucleus. PLpro is a domain of the larger NSP3 protein. In our studies a soluble PLpro form is assessed for its function in inhibiting IRF3 signaling. During a natural infection, the PLpro domain of NSP3 is localized on double membrane vesicles induced by coronaviruses during infection, with the PLpro domain in a cytoplasmic loop of NSP3. The effect of PLpro membrane localization during infection compared to the soluble form used here and its role on the IRF3 pathway is currently under investigation. Understanding the role of PLpro in SARS-CoV pathogenesis is critical to fully understand due to its central role as one of the two proteases responsible for ORF1ab polyprotein cleavage and its role as an IFN antagonist. The combined enzymatic activities of PLpro make it a highly attractive target for therapeutic inhibitor development.

PLpro is one of the two virally encoded proteases that are necessary for SARS-CoV to cleave the coronavirus ORF1A polyprotein into separate peptides that function in replication of SARS-CoV genomic RNA [26]. PLpro has protease activity *in vitro* and *in vivo* while also containing de-ubiquitination and de-ISGylation activity at the same active site [18]. We, and others have shown that PLpro inhibits IRF3 activation by blocking phosphorylation of IRF3 and subsequent induction of type I IFN gene transcription [16,23]. We hypothesized that PLpro could act to inhibit IRF3 using additional mechanisms due to its de-ubiquitinase activity. To this end, we analyzed the ability of PLpro to inhibit IRF3(5D), a phosphomimetic of IRF3 that replaces 5 serines (S) in the C terminus of IRF3 to aspartic acid (D), thus mimicking what phosphorylated and activated IRF3 [34]. IRF3(5D) is highly active when transfected into cells and readily induces interferon induced genes by binding to promoter regions and inducing transcriptional induction.

When IRF3(5D) was expressed in a cell, it induced the expression of IFNβ without any exogenous stimuli. We were able to show that PLpro inhibits IRF3(5D) dependent induction of IFNβ, suggesting that PLpro can also inhibit IRF3 at a step after phosphorylation. We further analyzed at which step PLpro inhibited the activity of IRF3(5D); from dimerization of IRF3 to binding to IFNβ gene promoter induction. We were able to demonstrate that PLpro does not inhibit IRF3(5D) dimerization in the cytoplasm, does not inhibit its nuclear import, or the ability to bind to the IFNβ promoter or other promoters of IRF3 inducible genes.

We hypothesized that IRF3(5D) is not correctly modified and thus unable to bind to the transcriptional machinery. It has been shown that IRF3 needs to be ubiquitinated to be transcriptionally active. Using a catalytically inactive PLpro mutant, we were able to show that the deubiquitinase function of PLpro is required for inhibition of IRF3(5D) dependent induction of IFNβ.

Viral inhibitors of innate immunity are critical for viral replication and pathogenesis. While many viral antagonists of IFN signaling have been identified, their mechanisms of action are unclear. In this study we have identified an additional role for PLpro in the inhibition of IRF3 signaling. Since no viral immune antagonist can fully inhibit its target, it is rational that a viral protein could evolve to inhibit the same pathway at multiple steps. SARS-CoV PLpro has been shown to inhibit IRF3 phosphorylation early in the type I IFN signaling pathway. Here, we demonstrate that SARS-CoV PLpro is able to inhibit IRF3 dependent type I IFN activation at a later stage, by deubiquitinating IRF3 and blocking its ability to induce IFNβ transcription. This additional activity demonstrates the important additional role that may be played by PLpro in the pathogenesis of SARS-CoV.
